# Carbon source–dependent capsule thickness regulation in *Streptococcus pneumoniae*

**DOI:** 10.3389/fcimb.2023.1279119

**Published:** 2023-11-29

**Authors:** Joel P. Werren, Nadja Mostacci, Ilche Gjuroski, Lalaina Holivololona, Lukas J. Troxler, Lucy J. Hathaway, Julien Furrer, Markus Hilty

**Affiliations:** ^1^ Institute for Infectious Diseases, Faculty of Medicine, University of Bern, Bern, Switzerland; ^2^ Graduate School for Cellular and Biomedical Sciences, University of Bern, Bern, Switzerland; ^3^ Department of Chemistry, Biochemistry and Pharmacy, University of Bern, Bern, Switzerland

**Keywords:** *Streptococcus pneumoniae*, monosaccharides, capsule, precursor, adherence, transcriptome

## Abstract

**Background:**

The polysaccharide capsule of *Streptococcus pneumoniae* plays a major role in virulence, adherence to epithelial cells, and overall survival of the bacterium in the human host. Galactose, mannose, and N-acetylglucosamine (GlcNAc) are likely to be relevant for metabolization in the nasopharynx, while glucose is the primary carbon source in the blood. In this study, we aim to further the understanding of the influence of carbon sources on pneumococcal growth, capsule biosynthesis, and subsequent adherence potential.

**Methods:**

We tested the growth behavior of clinical wild-type and capsule knockout *S. pneumoniae* strains, using galactose, GlcNAc, mannose, and glucose as carbon source for growth. We measured capsule thickness and quantified capsule precursors by fluorescein isothiocyanate (FITC)–dextran exclusion assays and ^31^P-nuclear magnetic resonance measurements, respectively. We also performed epithelial adherence assays using Detroit 562 cells and performed a transcriptome analysis (RNA sequencing).

**Results:**

We observed a reduced growth in galactose, mannose, and GlcNAc compared to growth in glucose and found capsular size reductions in mannose and GlcNAc compared to galactose and glucose. Additionally, capsular precursor measurements of uridine diphosphate-(UDP)-glucose and UDP-galactose showed less accumulation of precursors in GlcNAc or mannose than in glucose and galactose, indicating a possible link with the received capsular thickness measurements. Epithelial adherence assays showed an increase in adherence potential for a pneumococcal strain, when grown in mannose compared to glucose. Finally, transcriptome analysis of four clinical isolates revealed not only strain specific but also common carbon source-specific gene expression.

**Conclusion:**

Our findings may indicate a careful adaption of the lifestyle of *S. pneumoniae* according to the monosaccharides encountered in the respective human niche.

## 1 Introduction

**Figure 1:**
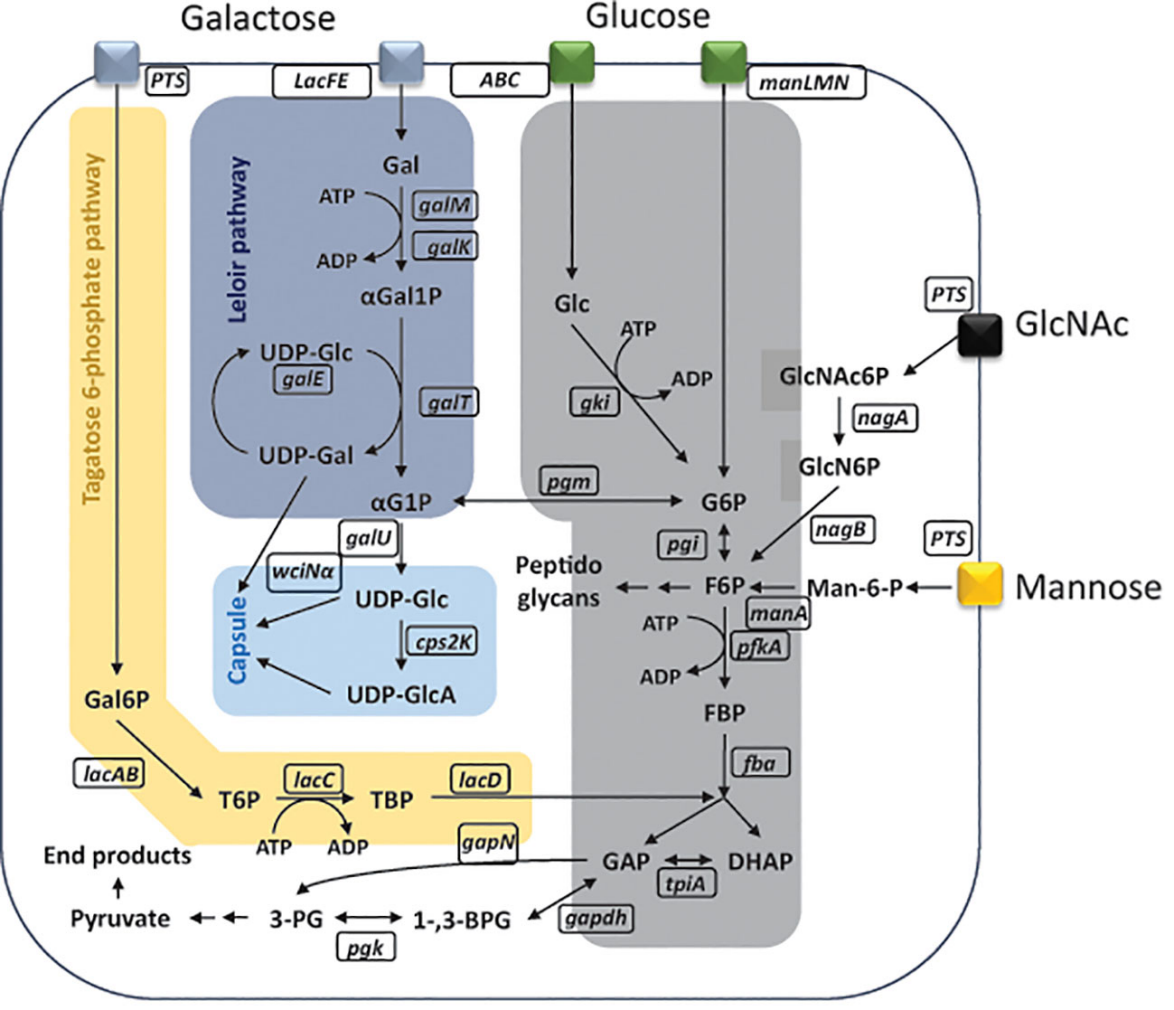
Schematic representation of metabolic pathways inside *Streptococcus pneumoniae*. Monosaccharide uptake phosphotransferase transporter (manLMN); phosphoenolpyruvate phosphotransferase (PTS) transporter; glucose (Glc); glucose kinase (gki); adenosine triphosphate (ATP); adenosine diphosphate (ADP); glucose-6-phosphate (G6P); glucose-6-phosphate isomerase (pgi); fructose-6-phosphate (F6P); 6-phosphofructokinase (pfkA); fructose-biphosphate (FBP); fructose-bisphosphate aldolase (fba); dihydroxyacetone phosphate (DHAP); glyceraldehyde-3-phosphate (GAP); triosephosphate isomerase (tpiA); glyceraldehyde-3-phosphate dehydrogenase (gapdh); 1,3-bisphosphoglycerate (1-,3-BPG); non-phosphorylating glyceraldehyde-3-phosphate dehydrogenase (gapN); reduced nicotinamide adenine dinucleotide phosphate (NADPH); nicotinamide adenine dinucleotide phosphate (NADP+); phosphoglycerate kinase (pgk); 3-phosphoglycerate (3-PG); alcohol dehydrogenase (adh); L-lactate oxidase (lctO); lactate dehydrogenase (ldh); α-glucose-1-posphate (αG1P); uridine diphosphate glucose (UDP-Glc); uridine diphosphate glucuronic acid (UDP-GlcA); UDP-glucose 6-dehydrogenase (cps2K); α-1,3-galactosyltransferase (wciNα); phosphoglucomutase (pgm); glucose-6-phosphate dehydrogenase (g6pd); 6-phosphogluconolactone (6PGL); galactose (Gal); galactose mutarotase (galM); galactokinase (galK); alpha-galactose-1-phosphate (αGal1P); galactose-1-phosphate uridylyltransferase (galT); alpha-glucose-1-phosphate (αG1P); glucose-1-phosphate uridylyltransferase (galU); uridine diphosphate galactose (UDP-Gal); UDP-glucose 4-epimerase (galE); galactose-6-phosphate (Gal6P); tagatose-6-phosphate (T6P); tagatose-6-phosphate kinase (lacC); tagatose-1,6-diphosphate (TBP); D-tagatose-1,6-diphosphate aldolase (lacD); N-acetylglucosamine-6-phosphate (GlcNAc6P); alpha-N-acetylgalactosaminidase (nagA); glucosamine-6-phosphate (GlcN6P); glucosamine-6-phosphate deaminase (nagB); mannose-6-phosphate (Man-6-P); mannose-6-phosphate isomerase (manA). Figure is adapted and modified from (Carvalho et al., 2011; Paixão et al., 2015b).

*Streptococcus pneumoniae* is a bacterium commonly colonizing the human nasopharynx but also able to cause invasive diseases (Brugger et al., 2016). It is a strictly fermentative bacterium, not able to gain energy from respiration, relying on carbohydrates for energy generation (Hoskins et al., 2001; Tettelin et al., 2001). Facing varying niches in the human host, *S. pneumoniae* is confronted with several nutritional environments such as the nasopharynx, dominated by mucins rich in galactose, N-acetylglucosamine (GlcNAc), mannose, or the blood, which is dominated by glucose (Aprianto et al., 2018; Hobbs et al., 2018). The bacterium possesses complex enzymatic machinery to process glycans into monosaccharides, ready for uptake via transporters, into the cell (Tettelin et al., 2001; Buckwalter and King, 2012; Hobbs et al., 2018). Its ability to uptake and process more than 30 different carbohydrates significantly contributes to the virulence of pneumococcus (Hava and Camilli, 2002; Obert et al., 2006). Monosaccharides can be taken up via a set of different transporters, mainly belonging to either adenosine triphosphate (ATP)–binding cassette (ABC) transporter superfamily or phosphoenolpyruvate phosphotransferase (PTS) system (Bidossi et al., 2012; Buckwalter and King, 2012). Glucose is the preferred carbon source for *S. pneumoniae* and transported inside the cell mainly via mannose-type PTS (manLMN), although uptake via ABC transporters is also hypothesized (Carvalho et al., 2011; Bidossi et al., 2012). Nasopharynx-occurring sugars such as galactose, GlcNAc, and mannose can also be transported via manLMN or other PTS systems (Bidossi et al., 2012). Following the uptake, glucose will be processed via Embden–Meyerhof–Parnas (EMP) pathway, the pentose phosphate pathway, or converted to UDP-glucose (UDP-Glc) and UDP-galactose (UDP-Gal) as a building block for the bacterial capsule (**Figure 1**) (Ramos et al., 2001; Härtel et al., 2012). If glucose is present in the environment of the pneumococcus, uptake and metabolism of other non-preferred sugars will be repressed through a process called carbon catabolite repression (CCR) (Beckwith and Zipser, 1970). This process is coordinated by the catabolite control protein A (CcpA) through binding catabolite response elements (CREs) in promoter regions of CCR-susceptible genes (Görke and Stülke, 2008; Carvalho et al., 2011). If glucose is not present, *S. pneumoniae* is able to grow on non-preferred sugars such as galactose, GlcNAc, and mannose (Paixão et al., 2015b). Galactose will be metabolized via Leloir pathway or tagatose-6-phosphate pathway, meanwhile mannose’s and GlcNAc’s phosphorylated products are isomerized to fructose-6-phosphate (F6P) (Afzal et al., 2014; Moye et al., 2014; Paixão et al., 2015b; Afzal et al., 2016a). The further processing will either continue through EMP for energy generation or biosynthesis of capsular precursors (**Figure 1**) (Paixão et al., 2015b).

The pneumococcal polysaccharide capsule counts as the most important virulence factor inside the host (Kadioglu et al., 2008). Pneumococcal serotyping, which identifies around 100 different capsule types, is based on the capsular composition (Geno et al., 2015; Ganaie et al., 2020). It has been shown that capsular biosynthesis is influenced by the nutritional environment that a pneumococcus is facing, like changes in oxygen or overall nutrient restricted conditions (Weiser et al., 2001; Hathaway et al., 2012). Furthermore, *S. pneumoniae’s* capsular size varies depending on the anatomical region in the host (Weiser et al., 1994; Weiser, 1998; Hammerschmidt et al., 2005; Arai et al., 2011). Also, the available carbon sources supplementing a chemically defined medium (CDM) have been shown to influence the production of capsular polysaccharides when comparing fructose and glucose (Weinberger et al., 2009; Troxler et al., 2019). The establishment of a thinner capsule most likely promotes epithelial adherence of pneumococcus by exposing surface proteins for cell attachment and also biofilm formation, meanwhile building a thicker capsule during systemic infection may allow evasion of destruction by complement-mediated opsonophagocytosis (Kim and Weiser, 1998; Kim et al., 1999; Hava et al., 2003; Hammerschmidt et al., 2005; Muñoz-Elías et al., 2008; Sanchez et al., 2011). Obviously, tight control of capsule expression is vital for pneumococcal survival in different niches, but the exact mechanisms behind capsule regulation are still unknown.

The genetic locus for capsule expression, the *cps* locus, is likely transcribed from a single promotor, upstream of *cpsA*, and in most of the known serotypes *cpsA*, *cpsB*, *cpsC*, and *cpsD* are highly conserved (Guidolin et al., 1994; Yother, 2011; Hathaway et al., 2012; Oliver et al., 2013). Genes downstream of *cpsD* encode enzymes involved in serotype-specific synthesis, polymerization, and export of capsular polysaccharides (Bentley et al., 2006; Yother, 2011). Not only the capsule locus but also an importance of the synthesis of capsular precursor UDP-glucose and UDP-galactose for capsule expression has been hypothesized and shown by others (Ramos et al., 2001; Carvalho et al., 2011). We have also shown that capsule precursor production and subsequent capsule biosynthesis depend on the carbon source present with the extreme of fructose-grown strains, presenting a nearly absent capsule compared to growth in glucose (Troxler et al., 2019). However, studies of capsule biosynthesis in regards to capsular thickness and capsule precursor production have not been done with nasopharynx relevant monosaccharides to date.

We therefore aimed to better understand the influence of different carbon sources on growth, capsular thickness, gene expression, and epithelial-cell adherence. To achieve our aim, we used a selection of clinical isolates, which enabled the investigation of strain-dependent and -independent features, together with their capsule knockout mutants (Δ*cps*).

## 2 Materials and methods

### 2.1 Bacterial strains and growth conditions

All the bacterial strains used in this project were collected from the Swiss National Reference Centre for Pneumococci. Strains 106.66 (6B), 103.57 (23F), 207.31 (15C), 208.41 (7F), and their capsule knockout mutants (Δ*cps*), with the exception of 207.31 for which no capsule knockout mutant could be made, were chosen for this project. Bacteria were streaked out on Columbia sheep blood agar (CSBA) plates, grown overnight at 37°C in a 5% CO_2_ atmosphere incubator and then “pre-grown” in 10-ml cultures inside tubes (Milian SA, CH) until they reached an optical density of a wavelength 600 nm (OD_600nm_) of 0.5. The tubes for pre-growth contained modified lacks medium (Adams and Roe, 1945) supplemented with glucose (20 mM). For the investigation of the influence of different pre-growth conditions on subsequent results, pre-growth in Lacks GlcNAc, galactose, or mannose (20 mM) was additionally done. Subsequently, the strains were grown in CDM with either glucose, GlcNAc, galactose, or mannose (5.5 mM) as described (Troxler et al., 2019). In brief, after centrifugation and washing, bacterial suspension was added to CDM (1:25 dilution) supplemented with a single carbon source at a concentration of 5.5 mM. Bacterial growth was tracked by measuring the OD_600nm_ using a Thermo Scientific Helios Epsilon UV-visible spectrophotometer with an adapter to allow measurement directly in the culture tubes.

The maximum density was calculated from the mean of the three replicates of the highest OD_600nm_ values. For the calculation of the maximal growth rate, time points were chosen during maximal growth (steepest slope) and the following formula was then used as described (Hirschmann et al., 2021):


$$\text{µ}\left[{\text{h}}^{-1}\right]\text{=(}\left({\text{ln OD}}_{600nm}\left({\text{t}}_{2}\right){\text{-ln OD}}_{600nm}\left({\text{t}}_{1}\right)\right)/\left({\text{t}}_{2}{\text{- t}}_{1}\right)$$

### 2.2 FITC-dextran exclusion assays for capsular thickness measurements

Capsule thickness was evaluated by fluorescence microscopy according to a method previously used and described (Weinberger et al., 2009; Schaffner et al., 2014). Cells were harvested by centrifugation at mid-log phase (OD of 0.15 or 0.25), washed, and resuspended in phosphate buffer saline (PBS). Cells were prepared for visualization on a microscope slide and imaged as described before (Hathaway et al., 2012). In brief, a total of 20-µg fluorescein isothiocyanate (FITC)–dextran was used for visualization. Microscopy was performed on a Zeiss Axio Imager M1 (Carl Zeiss, Germany) and 100× magnification with oil immersion. Around 10 fluorescence and brightfield pictures were taken and analyzed with free software ImageJ and UTHSCSA image tool. This procedure was repeated three times on different days, resulting in total around 30 pictures per strain and carbon source.

### 2.3 Intracellular metabolite extraction

Whole-cell EtOH extracts were prepared using a modified version of the method, described by *Ramos et al. (* Ramos et al., 2001). Bacterial cells were harvested after growth to mid-log phase in CDM by centrifugation and washed once each with ice-cold 0.8% NaCl and ice-cold H_2_O. They were then resuspended in ice-cold H_2_O and diluted with absolute EtOH at −20°C to a concentration of 60% EtOH. Cells were disrupted via vigorous shaking for 2h at 4°C. Cell debris was removed by ultracentrifugation using 3800 rpm for 5 min and ultrafiltration (Amicon 10-kDa centrifuge filter), then the solvent was evaporated under reduced pressure (25 mbar) using a rotavapor R-100 (Büchi, Switzerland). Dried samples were weighed and dissolved in 100 μl of nuclear magnetic resonance (NMR) buffer (20 mM MOPS, 5 mM NaOAc, and 1 mM ethylenediaminetetraacetic acid (EDTA) in D_2_O with 0.1% phosphonoacetic acid, and 0.1% Trimethylsilyl propionic acid, pH 7.4) transferred into 1.7-mm NMR microtubes (Bruker, USA) and submitted for measurement of ^31^P-NMR spectra.

### 2.4 NMR measurements

For the intracellular metabolite extracts, one-dimensional, decoupled ^31^P-NMR measurements were done. NMR data were collected on a Bruker Avance II (500 MHz) spectrometer equipped with a 1.7-mm triple-resonance (^1^H, ^13^C, ^31^P) microprobe head. ^31^P spectra were acquired using 4,096 scans with a spectral width of 40760.9 Hz, a recycling delay of 2 s, and an acquisition time of 0.402 s at a regulated temperature of 298 K. All experiments were recorded and processed using the TopSpin^®^ software (Bruker Biospin, Germany).

Peaks were assigned by either spiking samples with reference substances of phosphorylated metabolites or comparison to our previous study (Troxler et al., 2019). Concentrations of phosphorylated metabolites were obtained by measuring integrals of the assigned peaks for metabolites UDP-glucose, UDP-galactose, phosphoribosyl diphosphate (PRPP), ATP, and fructose-1,6-bisphosphate (FBP). These intervals were divided by the total integrated region, giving the percentage from the total integrals. Next, the percentage was multiplied with a specific conversion factor for every metabolite, which was obtained previously according to a classic calibration procedure, resulting in the concentration of metabolites. Finally, concentration was normalized by the dry weight of whole cell extract and was plotted as nanomole per milligram of dry weight.

### 2.5 Epithelial adherence assays with Detroit 562 cells

Bacterial cells were harvested at mid-log phase in CDM and put on ice for 30 min to stop growth. Bacteria were centrifuged and washed once with PBS, then resuspended in Dulbecco’s MEM (Thermo Fisher Scientific, Waltham, MA, USA). Suspension in MEM was diluted to reach target OD_600nm_ with a bacterial inoculum of 1.8 × 10^5^ bacteria/ml. Subsequently, 500µl of this inoculum and 500µl MEM were added to the wells of a 24-well plate containing Detroit 562 cells (seeding density: 3 × 10^5^ cells per well) from ATCC (American type Culture Collection, USA). The 24-well plates with bacteria and Detroit cells were incubated for 1h at 37°C in a 5% CO_2_ atmosphere incubator. Then, medium was removed and wells were washed 5 times with PBS. Cells were detached by adding 200 µl of trypsin-EDTA (0.05%, Thermo Fisher Scientific, USA) to each well and incubated for 10 min at 37°C. MEM (800 µl) was added and serial dilutions of bacteria per cell suspension were done. Ten microliter of dilutions was then streaked on CSBA plates and incubated overnight. Adherence of bacteria was calculated the following day. The data includes two measurements (technical duplicate) of three biological replicates (six data points in total) for each experiment. Tests used to determine statistical significance for each data set are indicated in the respective figure captions. Statistical analyses were performed using GraphPad Prism 9.5 software.

### 2.6 Bacterial RNA extraction

Bacteria were grown in CDM, supplemented with 5.5 mM carbon source, until mid-log growth phase, then RNA transcription was stopped with RNAprotect Tissue Reagent (Qiagen, Hilden, Germany) and stored at −20°C overnight. RNA extraction of three biological replicates was done with RNeasy kit (Qiagen, Hilden, Germany). Briefly, stored pellets were thawed and mixed with 700 µl of buffer RLT and 10 µl of β2-mercaptoethanol (Merck, Darmstadt, Germany) per 1 ml of buffer RLT (Qiagen, Hilden, Germany). This suspension was added to small tubes containing glass beads and lysed running one cycle of 5 min at 50 Hz in a Qiagen TissueLyser LT (Qiagen, Hilden, Germany). Lysed bacteria were centrifuged for 10 s at 14000 rpm and room temperature, supernatant was collected and further processed according to the manufacturers protocol. RNA quality and purity were checked with 2100 Bioanalyzer Instrument (Agilent Technologies, USA).

### 2.7 RNA sequencing

The quantity and quality of the purified total RNA was assessed using a Thermo Fisher Scientific Qubit 4.0 fluorimeter with the Qubit RNA BR Assay Kit (Thermo Fisher Scientific, Q10211) and an Advanced Analytical Fragment Analyzer System using a Fragment Analyzer RNA Kit (Agilent, DNF-471), respectively. Thereafter, 100ng of input RNA was depleted of ribosomal RNA using an Illumina Ribo-Zero plus rRNA Depletion Kit (Illumina, 20037135). Ribodepleted RNA were made into cDNA libraries using a TruSeq Stranded Total RNA Human/Mouse/Rat Kit (Illumina, 20020597) with IDT for Illumina TruSeq RNA UD Indexes (Illumina, 20022371). This was done according to the reference guide entitled “TruSeq Stranded Total RNA with Illumina Ribo-Zero Plus rRNA Depletion” (Illumina, Document #1000000092426 v01). The quantity and length of the cDNA libraries was determined using qubit and fragment analyzer as explained above. Equimolar-pooled cDNA libraries were paired-end sequenced using Illumina NovaSeq 6000 SP or S1 Reagent Kits v.15, 100 cycles (Illumina, 20028401 or 20028319, respectively) on an Illumina NovaSeq 6000 instrument. The quality of each sequencing run was assessed using Illumina Sequencing Analysis Viewer (Illumina version 2.4.7) and all base call files were demultiplexed and converted into FASTQ files using Illumina bcl2fastq conversion software v2.20. The quality control assessments, rRNA depletion, generation of libraries, sequencing and demultiplexing was carried out at the Next Generation Sequencing Platform, University of Bern. The raw reads data are available under PRJNA1004532.

### 2.8 Read mapping and visualization of RNA-sequencing data

The reads of the RNA-sequencing runs were first mapped to the 106.66 genome with Bowtie2 v. 2.3.4.1 (Liao et al., 2013). After mapping the number of counts for each gene was assessed with featureCounts v. 2.0.1 (Liao et al., 2013). All further analysis was performed in R v. 4.1.0. The log2 fold-change values with the according p-values (Wald test) were calculated with DESeq2 (Love et al., 2014). From all genes, 10% with the lowest mean expression were discarded from the analysis. Also, very short genes (10%) were removed. Then, the Benjamini and Hochberg adjusted *p*-values smaller than 0.01 were used. For the volcano plot visualizations, the genes with log2 fold changes above 2 (and below −2) and adjusted *p*-value smaller 0.01 were specifically indicated. For the Venn diagrams the genes with fold changes above 2 (and below −2) and adjusted *p*-values smaller 0.01 were counted and plotted.

## 3 Results

### 3.1 Bacterial growth shows carbon source-specific growth behavior

**Figure 2:**
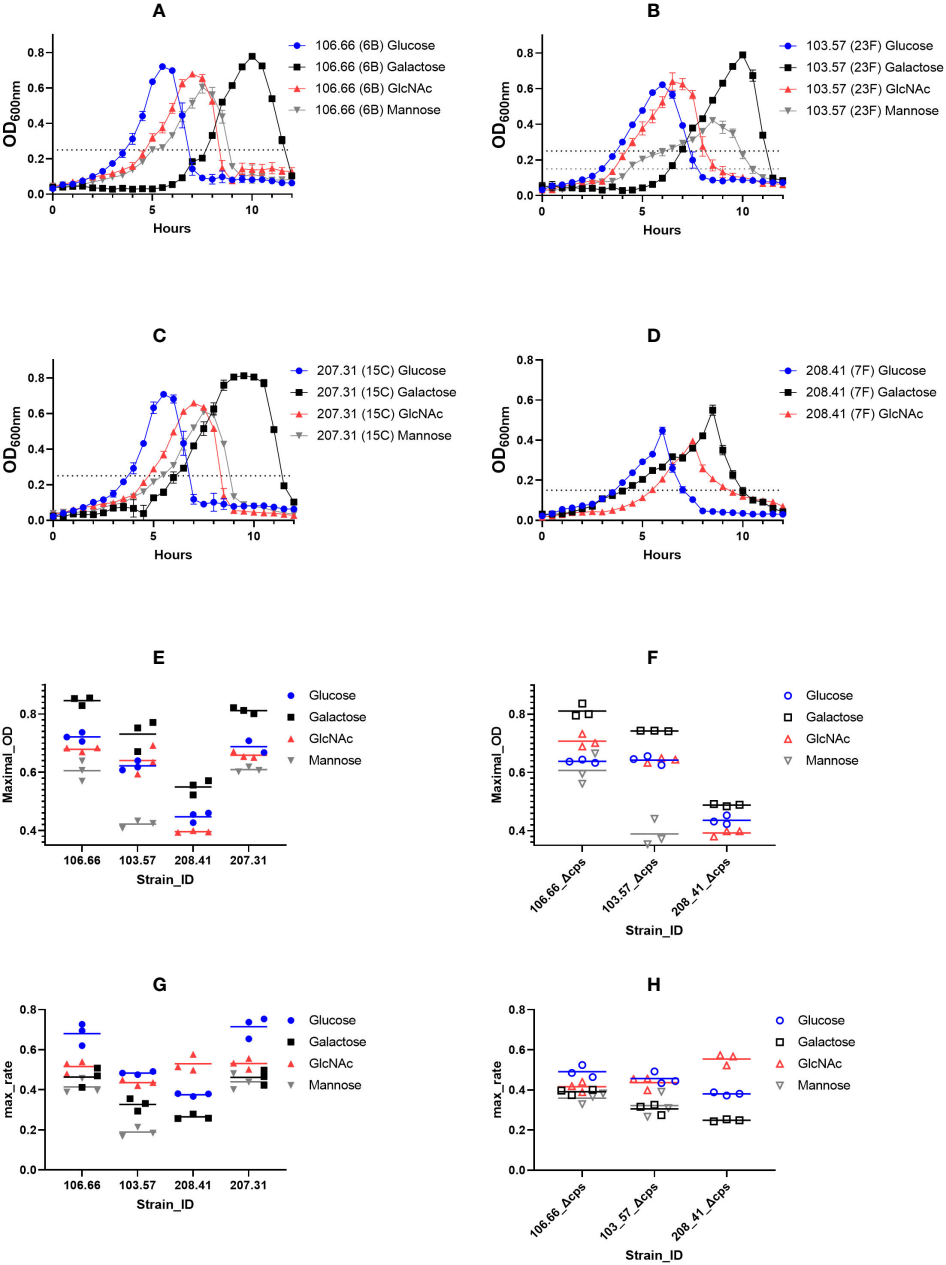
Growth measurements of pneumococcal strains in CDM, supplemented with different carbon sources. Strains 106.66 (serotype 6B) **(A)**, 103.57 (23F) **(B)**, 207.31 (15C) **(C)**, and 208.41 (7F) **(D)** were tested 3 times for growth behavior in four different conditions. Optical density (OD) during growth in 10 ml of CDM liquid cultures was measured in a spectrophotometer at 600_nm_ wavelength every 30 min over 12h. The mid-log OD_600nm_ values are indicated as dotted lines and were either 0.25 or 0.15. Maximal growth 600_nm_ values are shown for encapsulated **(E)** and non-encapsulated **(F)** strains. Also, maximal growth rates were calculated and shown for encapsulated **(G)** and non-encapsulated **(H)** strains. The strains 208.41 and 208.41Δcps did not grow in mannose.

We first tested four clinical isolates of *S. pneumoniae*, together with their capsule knockout mutants for their growth behavior (**Table 1**). Growth curves were analyzed using either glucose, galactose, GlcNAc, or mannose as carbon source (**Figures 2A-D** **;** **Supplementary Figure S1**). Strain-specific differences were noted as strains 106.66, 103.57, and 207.31, all reached similar maximal optical densities while this value was decreased for strain 208.41. Furthermore, strain 103.57 reached a lower maximal OD in mannose (OD_600nm:_ 0.4), compared to 106.66 and 207.31 (OD_600nm:_ 0.6) and strain 208.41 did not grow at all after 12h in this carbon source.

**Table 1:** List of pneumococcal strains used in this study.

ID	Description	Source
Wild types		
106.66	serotype 6B, MLST: 2244, IFIK	Clinical isolate (Mühlemann et al., 2003; Kronenberg et al., 2006)
103.57	serotype 23F, MLST: 507 IFIK	Clinical isolate (Mühlemann et al., 2003; Kronenberg et al., 2006)
207.31	serotype 15C, MLST: 199, IFIK	Clinical isolate (Mühlemann et al., 2003; Kronenberg et al., 2006)
208.41	serotype 7F, MLST: 191, IFIK	Clinical isolate (Mühlemann et al., 2003; Kronenberg et al., 2006)
Capsule knockout mutants		
106.66Δcps	106.66Δ*cps:.kan-rpsL* ^+^	Hathaway et al. (2012)
103.57Δcps	103.57Δ*cps:.kan-rpsL* ^+^	Hathaway et al. (2012)
208.41Δcps	208.41Δ*cps:.kan-rpsL* ^+^	Hathaway et al. (2012)

MLST, multi-locus sequence type. Strains were isolated at the IFIK (Institute for Infectious Disease, Bern, Switzerland). Capsule knockout strains were previously received with the exception of 207.31 for which no capsule knockout mutant could be made.

However, we noted similarities as the OD_max_ values were highest and lowest in galactose and mannose for all strains, respectively (**Figure 2E**). Importantly, comparing OD_max_ values of wild-type (WT) and non-encapsulated strains of the same genomic background revealed an identical pattern with very similar OD_max_ values (**Figure 2F**). In addition, the maximum growth rate values were higher if grown in glucose or GlcNAc as compared to galactose and mannose (**Figure 2G**). This was also true for the non-encapsulated strains (**Figure 2H**). Adapting the pre-growth conditions to the identical carbon source did not change the nature of the growth curves (**Supplementary Figure S2**). This means that, although the lag phase changed, the maximal OD and growth rate values did not change significantly.

### 3.2 Capsular thickness reduction in GlcNAc and mannose

**Figure 3:**
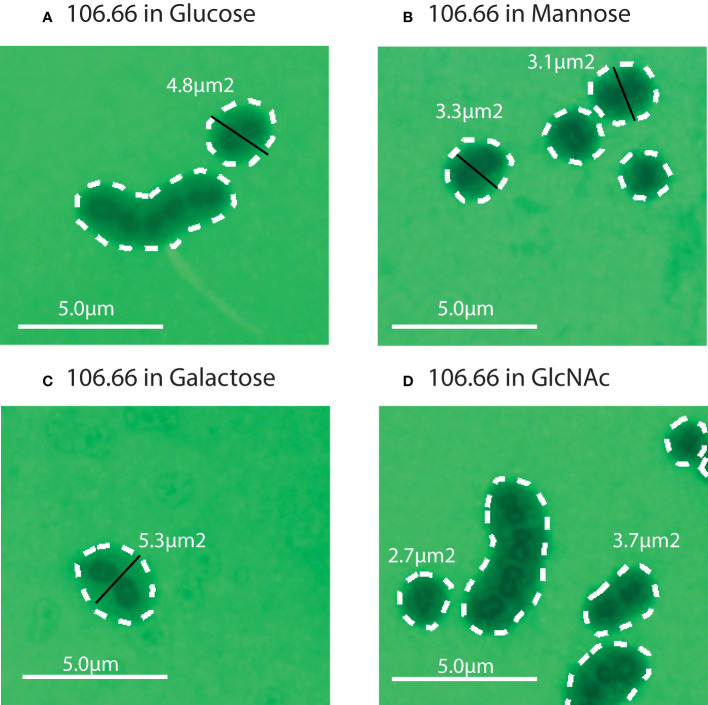
Four representative FITC microscopy pictures for the strain 106.66 in Glucose **(A)**, Mannose **(B)**, Galactose **(C)**, and GlcNAc **(D)**. The strain 106.66 was grown until mid-log growth phase which was 0.25 for all four carbon sources (see **Figure 2**). Diameters (in black) and area (white dotted lines) are indicated.

We then performed FITC-dextran exclusion assays to measure capsular thickness for all strains. Four representative pictures for the strain 106.66 in glucose, galactose, GlcNAc, and mannose are shown (**Figures 3A-D**).

**Figure 4:**
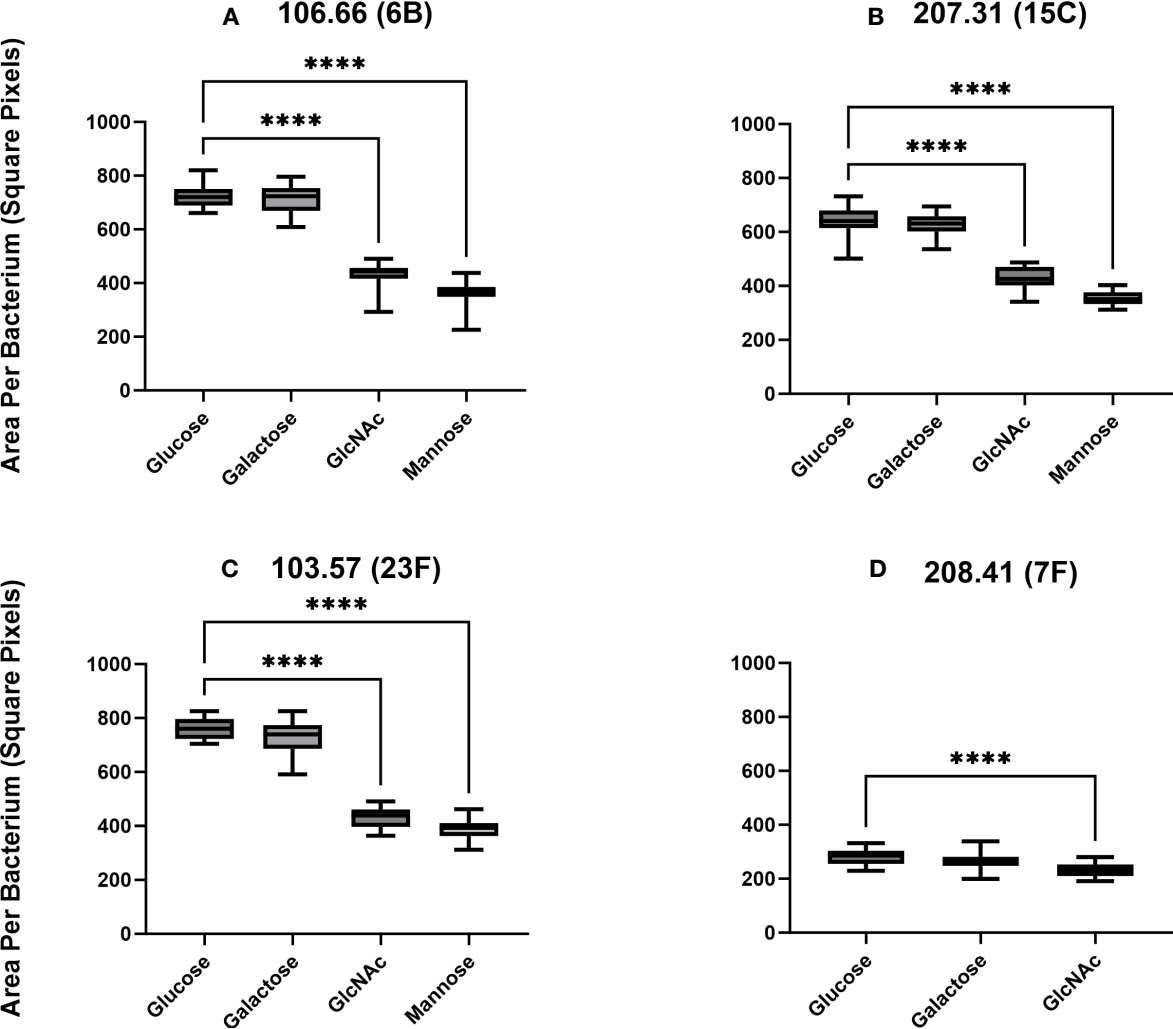
Capsular thickness measurements by FITC-dextran exclusion. Clinical strains 106.66 **(A)**, 207.31 **(B)**, 103.57 **(C)**, and 208.41 **(D)** were grown in CDM until mid-log growth phase and visualized under a fluorescence microscope. Ratios of capsule thickness following growth in GlcNAc versus glucose or mannose versus glucose for strains are (106.66: 0.6, 0.5); (207.31: 0.66, 0.54); (103.57: 0.57, 0.51); (208.41: 0.83). Strain 208.41 did not grow in mannose. (ordinary one-way ANOVA; ****p ≤ 0.0001). The data includes 10 pictures of three biological replicates (30 pictures in total) for each experiment.

We did not find a significant difference in capsule thickness between pneumococci grown in carbon sources galactose and glucose (*p* > 0.05) (**Figures 4A-D**). However, the capsular size differences between GlcNAc and mannose to glucose were significant (*p*< 0.0001). Capsular size reductions were about 34%–43% for GlcNAc and 46%–50% for mannose compared to glucose. The only exception was strain 208.41, which already established a very thin capsule in glucose, having only a 17% size reduction in GlcNAc (**Figure 4D**). Overall, thickness of 106.66, 103.57, and 207.31 was similar while this was very much less for 208.41 (serotype 7F). This was observed in all carbon sources. Importantly, if grown in glucose or mannose, capsule thickness was higher as compared to the non-encapsulated strains as shown in two representative pictures (**Supplementary Figure S3**).

We additionally tested strains 106.66 and 103.57 capsular thickness in CDM GlcNAc and mannose after pre-growth in those carbon sources compared to pre-growth in glucose (**Supplementary Figure S4**). We found no significant differences in capsular thickness between bacteria after pre-growth in glucose and CDM GlcNAc/mannose or after pre-growth in GlcNAc/mannose and CDM GlcNAc/mannose (*p* > 0.05). Therefore, the type of pre-growth did not affect capsule thickness differences.

### 3.3 Reduced accumulation of capsular precursors in CDM GlcNAc and mannose by ^31^P-NMR measurements

**Figure 5:**
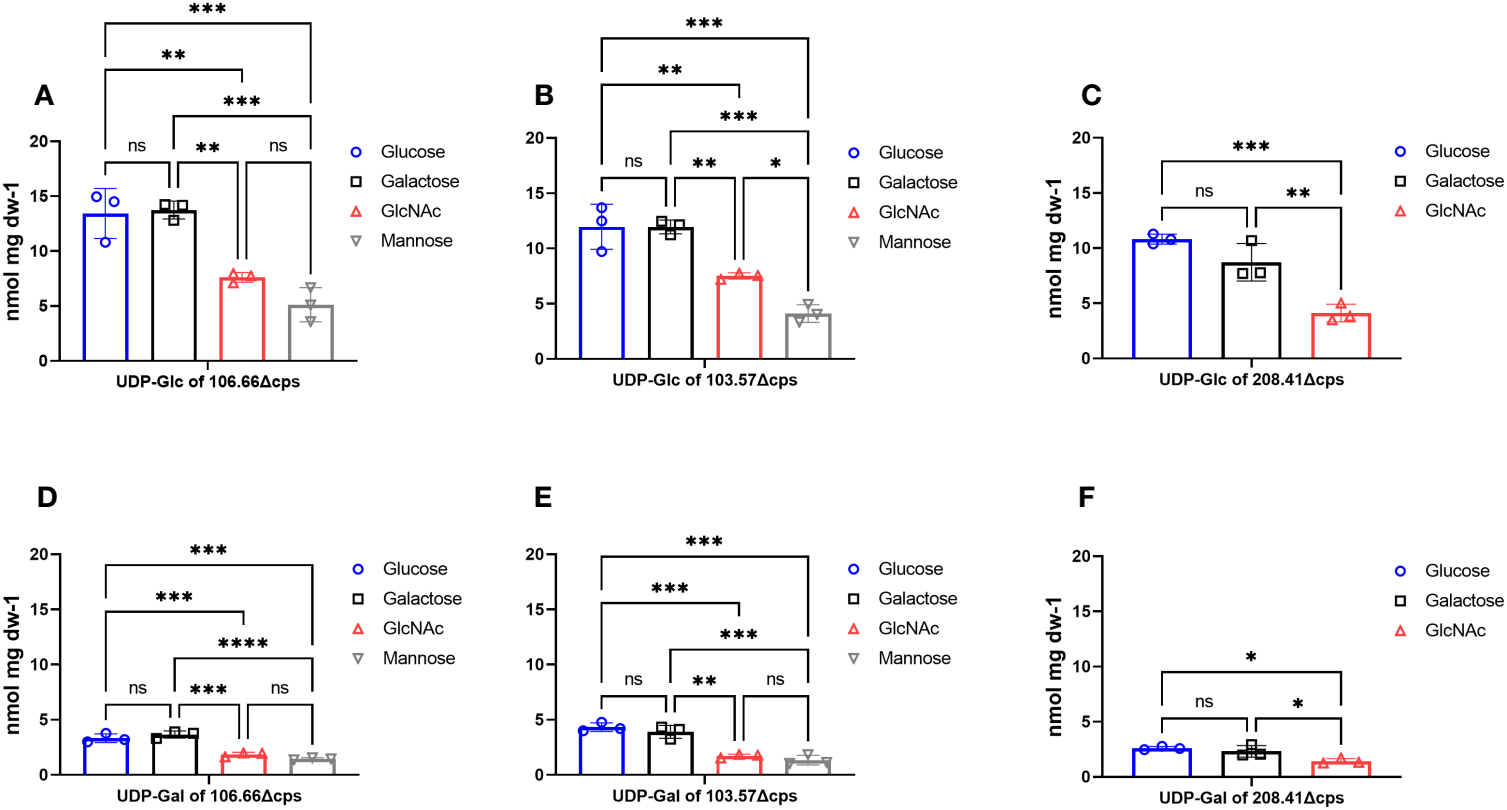
Measurements of phosphorylated UDP-sugars with ^31^P-NMR. Accumulation of intracellular phosphorylated metabolites UDP-glucose **(A–C)** and UDP-galactose **(D–F)** were measured in capsular knockout strains of 106.66Δcps, 103.57Δcps and 208.41Δcps with ^31^P-NMR. Strains were grown in CDM supplemented with either glucose (blue), galactose (black), GlcNAc (red) or mannose (gray) until mid-log phase. Intracellular metabolites were analyzed three times (*n* = 3) in samples after cold ethanol extraction and concentration was calculated as nmol per dry weight (dw) of sample. Strain 208.41Δcps did not grow in mannose. (ordinary one-way ANOVA; ns; *p* > 0.05; *p ≤ 0.05; **p ≤ 0.01; ***p ≤ 0.001; ****p ≤ 0.0001).

We next quantified capsular precursors alongside other phosphorylated intracellular metabolites by ^31^P-NMR measurements (**Supplementary Figure S5**). In order to measure accumulation of capsular precursors, capsular knockouts (Δ*cps*) are normally required since the encapsulated strains directly use UDP-sugars for building the capsule (Troxler et al., 2019). For both main capsular precursors UDP-glucose (**Figures 5A-C**) and UDP-galactose (**Figures 5D-F**), we found significant differences when strains were grown in GlcNAc (*p*< 0.01) or mannose (*p*< 0.001) compared to glucose and galactose. In agreement with the capsular thickness results, the quantities of precursors were also very low in mannose and in GlcNAc. We neither found differences in capsule size nor an accumulation of capsular precursors for glucose as compared to galactose (*p* > 0.05). Furthermore, we did not observe any significant differences for PRPP and ATP in strains 106.66Δ*cps* and 103.57Δ*cps* (**Supplementary Figure S6**) (*p*< 0.0001). In contrast, values for FBP were significantly higher in glucose and GlcNAc compared to galactose or mannose for both strains (*p*< 0.0001). A higher FBP accumulation was not found in the strain 208.41Δ*cps* (*p* > 0.05).

### 3.4 Epithelial adherence is increased for mannose

**Figure 6:**
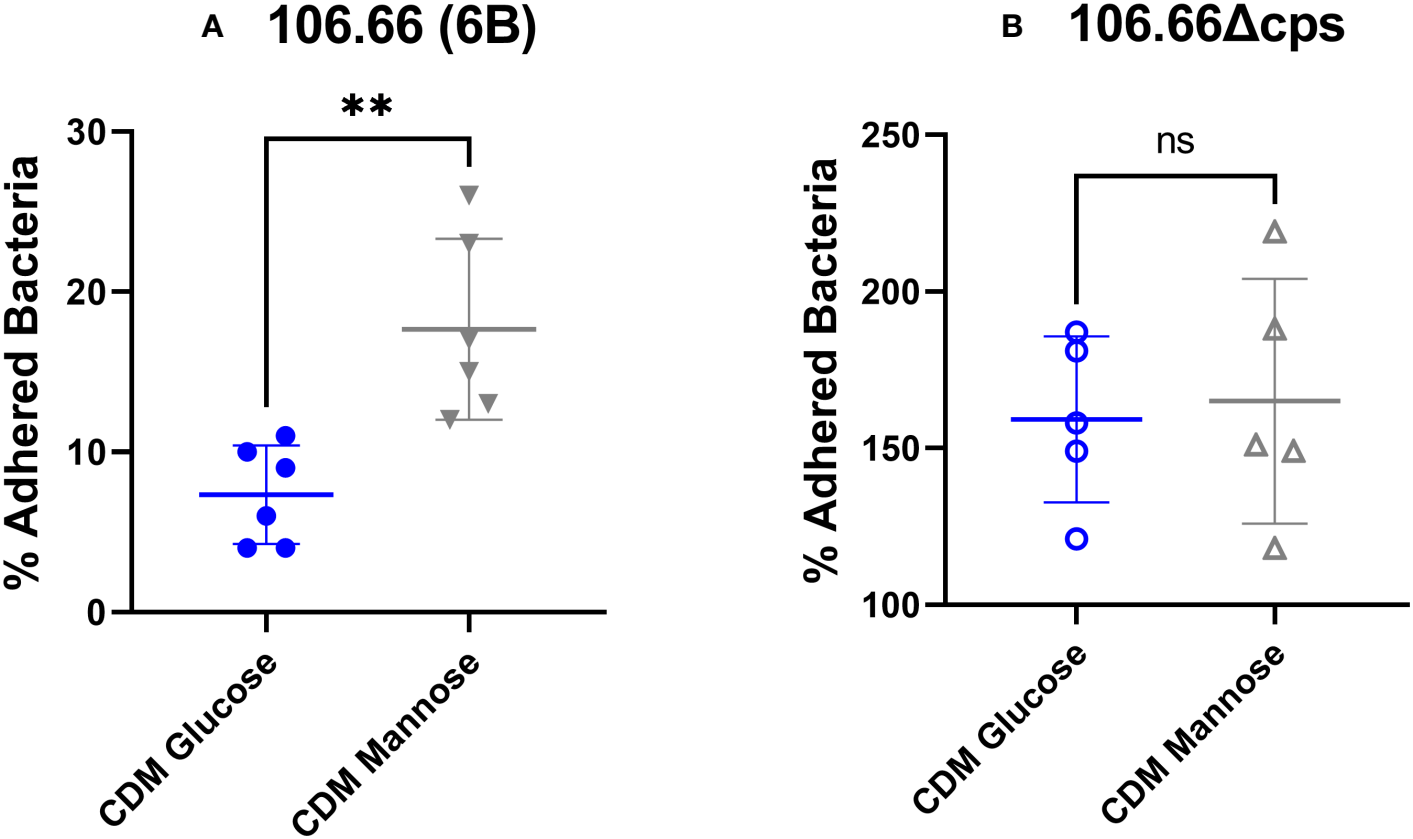
Adherence assay of strain 106.66 **(A)** and 106.66Δcps **(B)** in CDM with different carbon sources. Strains 106.66 **(A)** and 106.66Δcps **(B)** were grown in CDM supplemented with glucose or mannose and incubated with Detroit 562 cells. Adherence was measured by counting bacteria on CSBA plates after adherence and normalizing by original inoculum (Mann–Whitney test; **p ≤ 0.01). The data include two measurements (technical duplicate) of three biological replicates (six data points in total) for each experiment. For one biological replicate of (B), only one value rather than a technical duplicate has been received (five data points in total). ns, not significant.

A dynamic regulation of the capsule is necessary for pneumococcal survival inside the host in varying niches (Hammerschmidt et al., 2005; Weiser et al., 2018). Earlier work has shown that pneumococci with reduced capsular material adhere much better to epithelial cells than those with thicker capsules; meanwhile, a thicker capsule is required in blood environment for immune evasion (Kim and Weiser, 1998; Kim et al., 1999; Hava et al., 2003; Hammerschmidt et al., 2005; Muñoz-Elías et al., 2008; Sanchez et al., 2011). We decided to proceed with epithelial adherence assays comparing the two extremes: glucose and mannose in the serotype 6B strain 106.66. Indeed, 106.66 showed an increased adherence, when grown in mannose with around 18% of adhered bacteria of initial inoculum meanwhile in glucose this value was about 9% (**Figure 6A**) (*p*< 0.01). Using the non-encapsulated strain 106.66Δcps, the percentage of adhered bacteria increased to around 150% but no difference between glucose and mannose was noted anymore (**Figure 6B**).

### 3.5 Transcriptome analysis for all strains reveals differences in gene expression between carbon sources

**Figure 7:**
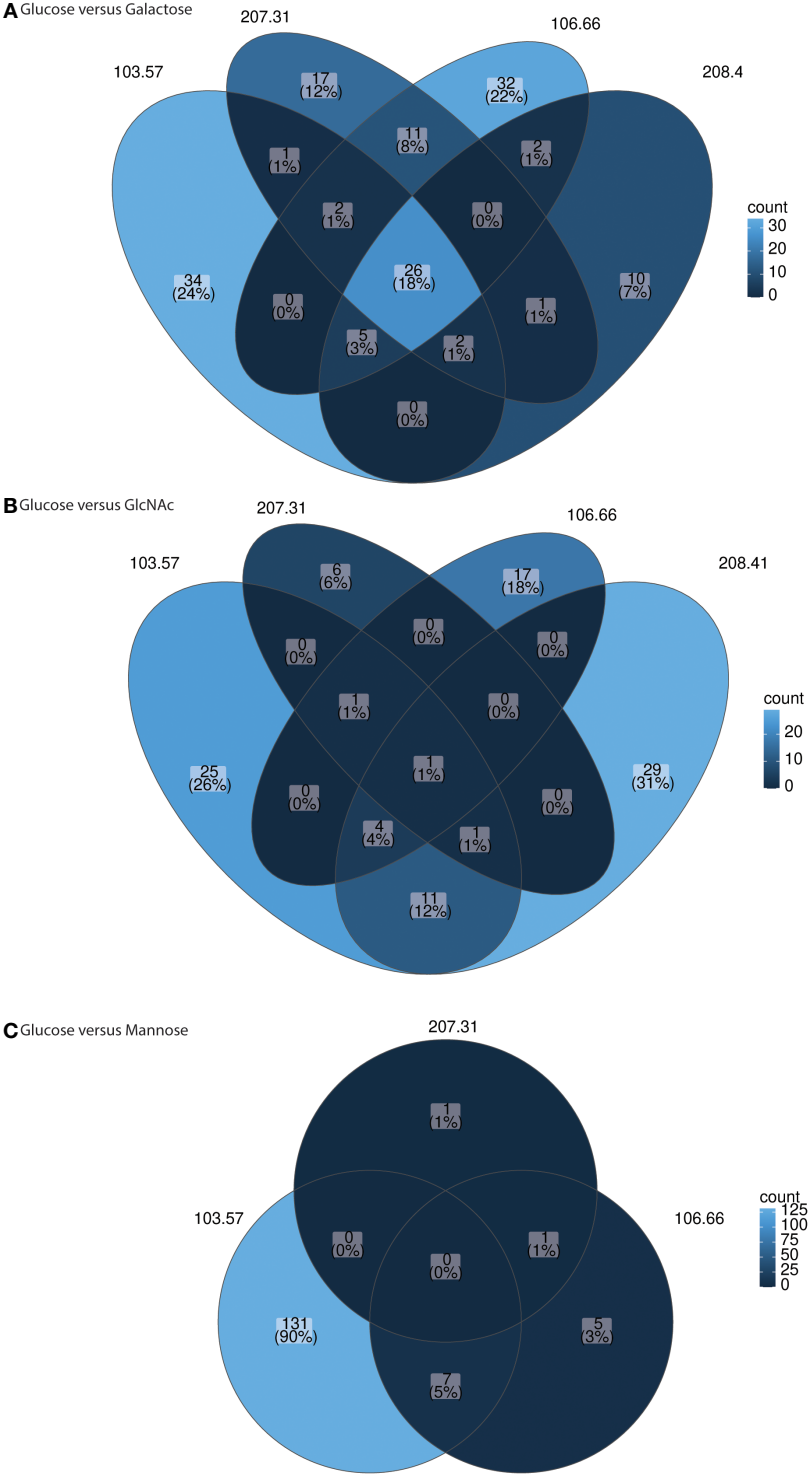
Venn diagrams for gene expression comparison glucose versus other carbon sources for individual strains. The genes with log2 fold changes above 2 (and below −2) and adjusted *p*-values smaller 0.01 were counted and plotted in the Venn diagrams. The comparisons glucose versus galactose **(A)**, glucose versus GlcNAc **(B)**, and glucose versus mannose **(C)** are shown for strain 106.66, 207.31, 103.57, and 208.41. The strain 208.41 did not grow in mannose. RNA sequencing was done for three biological replicates.

We subsequently investigated gene expression after growth in galactose, GlcNAc, or mannose in comparison to glucose. We received a total number of paired-reads per sample from 15.9 to 46.7 million paired-reads (average: 23.2 million paired-reads). On average, 27.0, 14.5, 15.5, and 27.7 reads were mapped to annotated features onto the pneumococcal reference genome (i.e., 106.66) for 106.66, 207.31, 103.57, and 208.41, respectively. Volcano plots for all strains for glucose versus galactose, glucose versus GlcNAc, and glucose versus mannose were received (**Supplementary Figures S7–S9**). In addition, under- and overexpressed genes with *p*< 0.01 were counted and plotted in three Venn diagrams (**Figures 7A-C**). We noted a high number of genes which were overexpressed in galactose for all strains (*n* = 26) (**Figure 7A**). If grown in galactose, this resulted in a strong upregulation of genes from the Leloir and tagatose pathway. Furthermore, upregulation of genes for PTS or ABC transporters were identified for galactose (**Supplementary Figure S7**). On the other hand, gene *gapN* (encoding the non-phosphorylating glyceraldehyde-3-phosphate dehydrogenase) was strongly upregulated in glucose for three strains. As for GlcNAc, we identified *nagA* (N-acetylglucosamine-6-phosphate deacetylase) and *nagB* (glucosamine-6-phosphate deaminase) being up- and *glmS* being downregulated in the majority of strains (**Supplementary Figure S8**). Finally, if grown in mannose, this led to the upregulation of *nanA* in two strains (**Supplementary Figure S9**). In general, only few changes were noted if grown in GlcNAc and mannose as compared to glucose (**Figures 7B, C**).

Importantly, we did not note any gene expression differences in the capsule region. Therefore, the expression of capsule genes does not explain the differences found for capsule thickness.

## 4 Discussion

This study shows how *S. pneumoniae* regulates its capsule biosynthesis according to the carbon source environment by comparing the nasopharynx carbon sources galactose, GlcNAc, and mannose to the blood carbon source glucose in four clinical isolates. We chose these particular carbon sources, since previous studies showed, that *S. pneumoniae* was able to grow on mucins and upon growth, gene expression in D39 showed strong upregulations of genes for metabolization of galactose, GlcNAc and mannose (Yesilkaya et al., 2008; Paixão et al., 2015b). It has also been shown by others that capsular thickness is regulated in response to environment and differs throughout changing niches (Hammerschmidt et al., 2005).

Pneumococcal growth is regulated by the carbon catabolite control (CCR) via transcriptional regulator CcpA (Carvalho et al., 2011; Al-Bayati et al., 2017). In agreement with previous growth results from in *S. pneumoniae* D39 (Paixão et al., 2015b), with the exception of the strain 208.41, the maximum growth rates were the highest if grown in glucose in our study. It has been described that glucose is the preferred carbon source if 13 or 31 mM of glucose were used for the growth of D39 (Paixão et al., 2015b). We used a concentration of 5.5 mM, which is probably similar to that found in the blood but the growth rate finding was similar as shown for D39. The growth in galactose was slower but the highest maximal OD values were observed in this carbon source. Interestingly, the strain 208.41 (7F) generally grew “worse” and not at all in mannose as compared to other strains. It could be that the strain would grow after all, but we just do not see this with our experimental design during the 12h of growth (e.g., there is a very long lag time). However, at this stage, we do not have an explanation for the growth behavior of 208.41.

It has been shown that the production of polysaccharide capsule in *S. pneumoniae* interferes with growth in nutrient-limiting conditions probably due to the fact that the biosynthesis of the capsule is in competition with the central metabolism for energy, that is, ATP (Hathaway et al., 2012). Therefore, we included WT and non-encapsulated strains for the growth assay. Based on the chosen growth conditions of this study, we could not see that the synthesis of the capsule influenced growth as the growth curves of WT and non-encapsulated strains were remarkably similar. This probably reflects the fact that, though defined, many of the compounds used are in excess rather than limiting in our CDM.

We then hypothesized that an increased maximum growth rate is correlated with an increase of the capsule thickness as there might be an accumulation of capsule precursor molecules which would subsequently lead to a thicker capsule. Indeed, it has been shown that strains grown in BHI+FCS [brain heart infusion and fetal calf serum (FCS)] showed overall better bacterial growth and more capsule production than growth in minimal lacks medium (Hathaway et al., 2012). However, in this study, capsule size thickness of the strains grown in glucose as compared to galactose did not differ despite the decreased maximum growth rate for the latter. This is probably due to the fact that galactose has its own pathway (Leloir) for building capsular polysaccharides, which works very efficiently in all four clinical isolates (Paixão et al., 2015b; McLean et al., 2020). Alternatively, it is known that additional molecules of ATP are generated (due to acetate kinase activity) if galactose is used as a carbon source, which is potentially an advantage for the synthesis of the capsule during the metabolism of slowly metabolizable sugars like galactose (Paixão et al., 2015a). However, we found significant size reductions in capsular thickness of our pneumococcal strains grown in GlcNAc and mannose as compared to glucose.

We have previously shown that the quantification of UDP-glucose and UDP-galactose can only be done in capsule knock as the precursor do not accumulate in WT strains (Troxler et al., 2019). By measuring the quantities of UDP-glucose and UDP-galactose in the capsule knockout strains, we were able to confirm the microscopy findings. This was due to reduced quantities of the two capsule precursors in mannose and GlcNAc as compared to glucose for strains 106.66Δ*cps*, 103.57Δ*cps*, and 208.41Δ*cps*. We speculate that the efficiency of the biosynthesis of the capsular precursors may depend on possible bottlenecks in enzymatic reactions responsible for the import or metabolization of the respective monosaccharide (**Figure 1**). Related to this, it has been shown for D39 that there is an accumulation of α-Gal6P, GlcNAc6P, and Man6P, which indicate metabolic bottlenecks in the metabolism of galactose, GlcNAc, and mannose, respectively (Paixão et al., 2015a; Paixao et al., 2015b). Interestingly, in *Corynebacterium glutamicum*, it has been shown that overexpression of manA alleviated the accumulation of Man6P and improved mannose catabolism indicating that this is indeed a bottleneck (Sasaki et al., 2011). As for galactose and as mentioned above, the biosynthesis of the capsule precursors follows the Leloir pathway and, therefore, a potential bottleneck resulting in the accumulation of Gal6P in the tagatose pathway should have no effect on the biosynthesis of the capsule. In our study, we were not able to quantify α-Gal6P, GlcNAc6P, and Man6P probably due to the fact that we measured the phosphorylated metabolites during different time points as compared the study with D39 (Paixão et al., 2015a; Paixao et al., 2015b).

We also measured several other phosphorylated metabolites and found differences for FBP, which was increased in glucose and GlcNAc for which the maximum growth rates were high. An increase in FBP has also been found for D39 and it has been mentioned that it is well documented that FBP accumulates to higher amounts during the catabolism of fast metabolizable sugars as glucose than less preferred carbohydrates (Paixão et al., 2015a). However, the reasons for the accumulation of this metabolite are diverse and not entirely known.

In this study, we have been investigating monosaccharides, which are part of the major host-derived glycans, including the N- and O-linked glycans (Hobbs et al., 2018). The latter are often part of mucins and are composed of GlcNAc, N-acetylgalactosamine (GalNAc), N-acetylneuraminic acid (NeuNAc), galactose, and fucose. In our study, we found that the metabolization of mannose leads to a thinner capsule and increased adherence probably due to a better exposure of surface proteins for adhesion when the capsule is thinner (Paterson and Orihuela, 2010; Novick et al., 2017). Therefore, we hypothesize that the pneumococcus is able to regulate its capsule thickness while degrading and metabolizing mannose from, for example, N-linked glycans and, while doing so, increases its ability to adhere. However, there could be alternative explanations for the increased adherence. It was previously reported that increased chain length correlates with better adherence to human epithelial cells *in vitro* (Rodriguez et al., 2012). We did not systematically address if there are differences in the frequency of longer chains for the different monosaccharides. However, we did not find a difference in adherence for the non-encapsulated strains growth in glucose as compared to galactose, which indicates that capsule thickness differences are indeed responsible for the found difference for the WT strains.

It has been shown that galactose plays a key role in *S. pneumoniae* for colonization, adherence, biofilm formation, and infection (Blanchette et al., 2016; Al-Bayati et al., 2017; Andreassen et al., 2020; McLean et al., 2020). However, studies have mostly focused on the laboratory strain D39. In this study, we also present transcriptome data for the different carbon sources for the four different clinical strains. This allows the identification of the common but also individual transcriptome changes (i.e. transcriptional changes of the accessory genome). The latter may lead to *in-vivo* advantage for certain strains over others, but this has to be followed up in future studies. Common to all the strains, many of the identified, upregulated genes in galactose are likely involved in galactose uptake and processing, for example, the Leloir and tagatose pathway as shown in D39 (Afzal et al., 2014; Paixão et al., 2015b; Afzal et al., 2016b).

For GlcNAc as carbon source, it has been shown that this sugar is taken up by PTS transporters before being processed by the α-N-acetylgalactosaminidase (NagA) and the glucosamine-6-phosphate deaminase (nagB) (Bidossi et al., 2012; Moye et al., 2014). *Afzal et al.* (Afzal et al., 2016a) revealed that GlcNAc leads to the expression of *nagA* and *nagB* in D39 and proved both of them essential for pneumococcal growth on this sugar. Therefore, as expected, we also observed upregulation of *nagA* and *nagB* in all four strains when grown in GlcNAc. Furthermore, RNA data of glucose versus GlcNAc presented a very strong upregulation in glucose of gene *glmS*. GlmS encodes the glutamine-F6P aminotransferase, the first important enzyme for pneumococcal peptidoglycan synthesis and belongs to the N-acetylglucosamine (NAG) regulon together with *nagA* and *nagB* (Carvalho et al., 2011; Moye et al., 2014). Consistent with our data, Moye et al. (Moye et al., 2014) also showed an upregulation of *nagA* and *nagB*; meanwhile, a downregulation of *glmS* when *S. mutans* was grown in presence of GlcNAc.

Overall, the transcriptomic data do not provide information about the mechanism of capsule thickness regulation. Nevertheless, we think this data is important, as it excludes a role of different expression of the capsule genes and shows the broad variability of differently regulated genes among strains overall.

## 5 Conclusion

Overall, we have shown that *S. pneumoniae* is influenced by the available carbon source and establishes a different growth pattern according to the sugar. The chosen carbon source also directly translates to the biosynthesis of different quantities of capsule precursors. The consequence of the latter is the varying capsule thickness. The first step toward pneumococcal infection resulting in adhering to epithelial cells of the nasopharynx was facilitated with a thinner capsule, which we were also able to show. Transcriptome analysis for all our strains showed consistent carbon-source-specific upregulation of certain gene clusters and genes, important for the uptake and metabolism of the sugars used and also common and unique clusters across all three nasopharynx carbon sources. It will be interesting to follow up if certain pneumococcal strains are better adapted for the metabolization of different carbohydrates, adherence, and virulence as compared to others. This may explain why certain pneumococcal clones are more successful than others and, therefore, more attention to the expression and relevance of the accessory genome is recommended.

## Data availability statement

The datasets presented in this study can be found in online repositories. The names of the repository/repositories and accession number(s) can be found below: https://www.ncbi.nlm.nih.gov/genbank/, PRJNA1004532.

## Author contributions

JW: Formal analysis, Methodology, Validation, Writing – original draft, Conceptualization, Investigation, Software. NM: Data curation, Methodology, Resources, Visualization, Formal analysis, Investigation, Software, Writing – review & editing. IG: Methodology, Investigation, Writing – review & editing. LH: Methodology, Investigation, Writing – review & editing. LT: Methodology, Investigation, Writing – review & editing. LJH: Data curation, Methodology, Writing – review & editing. JF: Methodology, Investigation, Writing – review & editing. MH: Conceptualization, Funding acquisition, Methodology, Project administration, Supervision, Writing – review & editing, Investigation.
