# Supplementary material for: Carbon source–dependent capsule thickness regulation in Streptococcus pneumoniae
Source: Front Cell Infect Microbiol. 2023 Nov 29;13:1279119. doi: 10.3389/fcimb.2023.1279119 (PMC10716237; doi:10.3389/fcimb.2023.1279119)
Supplement: Supplementary file 1 [file DataSheet_1.docx]

**Supplementary Figures**

**Figure S1: Growth curves of WT versus capsule knock out strains.** The growth curves for 106.66 (A), 106.66∆cps (B), 103.57 (C), 103.57∆cps (D), 208.41 (E) and 208.41∆cps (F) in glucose (Glc), galactose (Gal), GlcNAc and mannose (Man) are illustrated. Glucose was used as the carbon source for the pre-growth (pre) condition.

**Figure S2: Investigation of the influence of different pre-growth conditions.** The growth curves for 106.66 (A and B), 106.66∆cps (C and D), 103.57 (D and E), 103.57∆cps (F and G), in glucose (Glc), galactose (Gal), GlcNAc and mannose (Man) are illustrated. The different pre-growth (pre) conditions are indicated.

**Figure S3: Fluorescein isothiocyanate (FITC)-Dextran Exclusion Assay microscopy pictures of 106.66Δcps grown in Glucose (top) and Mannose (bottom).** Cells were harvested at mid-log phase and capsule thickness was measured by FITC-microscopy.

**Figure S4: Analyses of Fluorescein isothiocyanate (FITC)-Dextran Exclusion Assay microscopy pictures of strains 106.66 and 103.57 with varying Pre-Growth Conditions.** Both strains were grown in CDM containing GlcNAc or mannose after specific pre-growth medium Lacks in either glucose, GlcNAc or mannose and visualized under a fluorescence microscope. (ordinary one-way ANOVA; ns; p > 0.05)

**Figure S5: Representative NMR Spectra of 106.66∆cps.** NMR example spectra of 106.66 is shown from carbon sources glucose, galactose, GlcNAc, mannose. Metabolites of interest UDP-glucose and UDP-galactose are marked.

**Figure S6: Measurements of phosphorylated Metabolites with ^31^P-NMR.** Accumulation of intracellular phosphorylated metabolites was measured in capsular knock-out strains of 106.66, 103.57 and 208.41 with ^31^P-NMR. Strains were grown in CDM, supplemented with a single carbon source and grown until mid-log phase. Intracellular metabolites were analyzed in samples after cold ethanol extraction and concentration was calculated as nmol per dry weight of sample. 208.41∆cps did not grow in mannose. (ordinary one-way ANOVA with p > 0.05 (ns), p ≤ 0.05 (*), p ≤ 0.01 (**), p ≤ 0.001(***) and p ≤ 0.0001(****))

**Figure S7: Volcano plots for glucose versus galactose.** Four different comparisons for strain 106.66, 207.31, 103.57 and 208.41 (glucose versus galactose) are shown. The genes with fold changes above 2 (and below -2) and adjusted p-value smaller 0.01 are indicated in red. Importantly, all the reads of the RNA sequencing runs were mapped to the 106.66 genome.

**Figure S8: Volcano plots for Glucose versus GlcNAc** Four different comparisons for strain 106.66, 207.31, 103.57 and 208.41 (glucose versus GlcNAc) are shown. The genes with fold changes above 2 (and below -2) and adjusted p-value smaller 0.01 are indicated in red. Importantly, all the reads of the RNA sequencing runs were mapped to the 106.66 genome

**Figure S9: Volcano plots for Glucose versus Mannose** Four different comparisons for strain 106.66, 207.31, 103.57 and 208.41 (glucose versus mannose) are shown. The genes with fold changes above 2 (and below -2) and adjusted p-value smaller 0.01 are indicated in red. Importantly, all the reads of the RNA sequencing runs were mapped to the 106.66 genome

**Figure S1: Growth curves of WT versus capsule knock out strains**

**Figure S2: Different pre-growth conditions**

**Figure S3: FITC picture of 106.66Δcps in Glucose (top) and Mannose (bottom)**

******

**Figure S4: Fluorescence Microscopy of strains 106.66 and 103.57 with varying Pre-Growth Conditions.**

**Figure S5: Representative NMR Spectra of 106.66∆cps.**

**UDP-Glucose**

**UDP-Galactose**


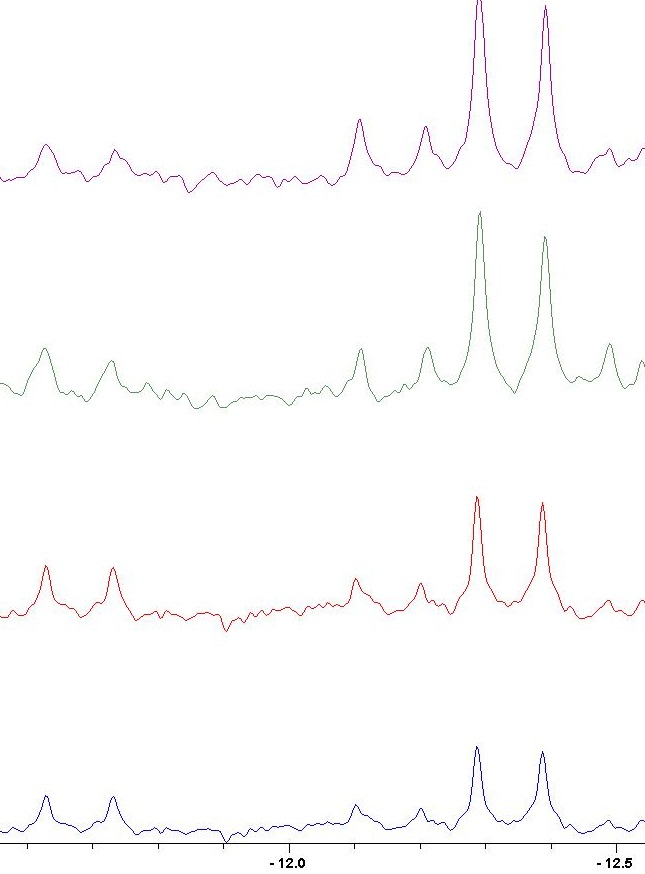
**ppm**

**CDM Mannose**

**CDM GlcNAc**

**CDM Glucose**

**CDM Galactose**

**Figure S6: Measurements of phosphorylated Metabolites with ^31^P-NMR.**


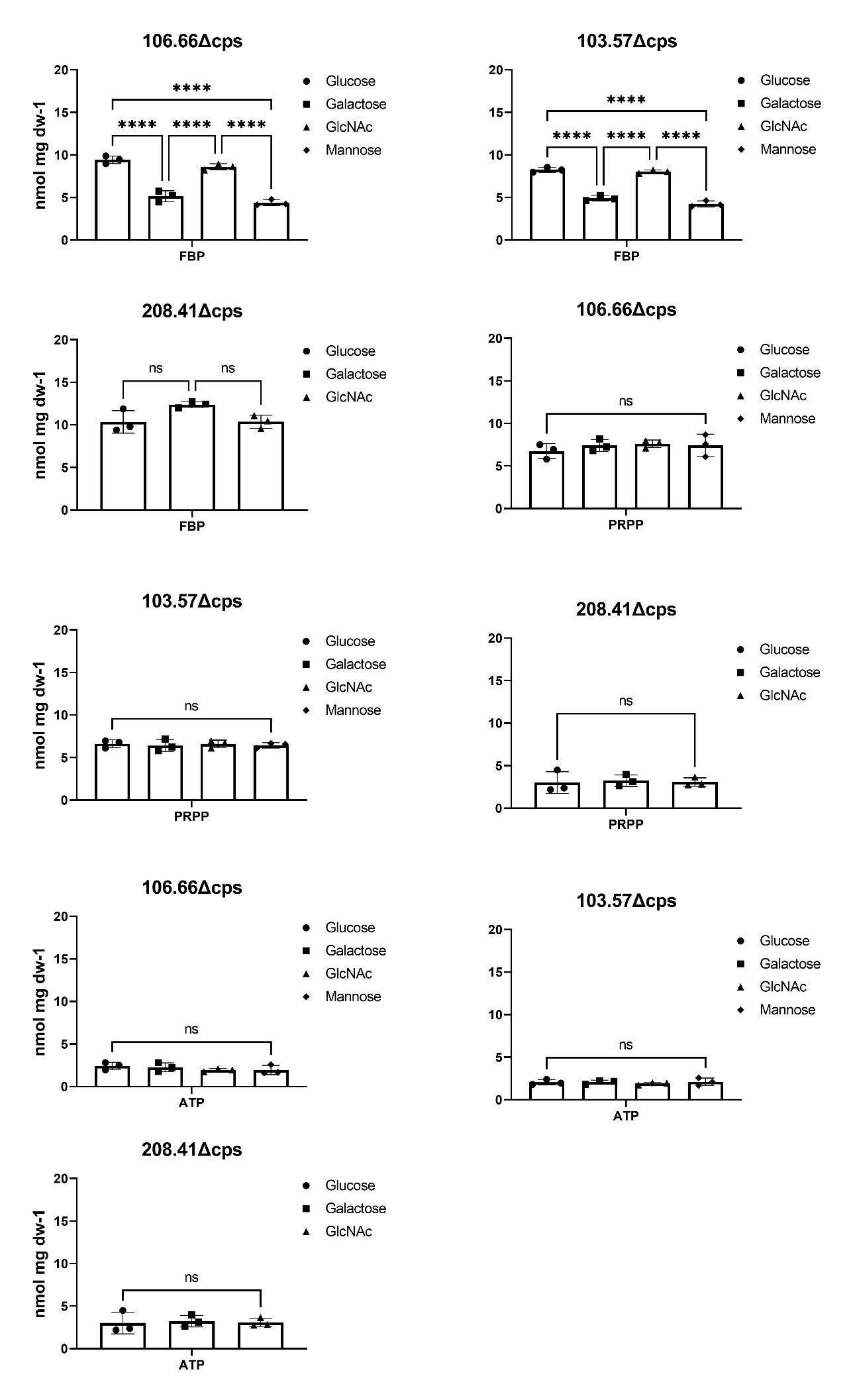


**Figure S7: Volcano plots for Glucose versus Galactose**
**Figure S8: Volcano plots for Glucose versus GlcNAc**

**Figure S9: Volcano plots for Glucose versus Mannose**
